# Infection free “resisters” among household contacts of adult pulmonary tuberculosis

**DOI:** 10.1371/journal.pone.0218034

**Published:** 2019-07-18

**Authors:** Vidya Mave, Padmapriyadarshini Chandrasekaran, Amol Chavan, Shri Vijay Bala Yogendra Shivakumar, Kavitha Danasekaran, Mandar Paradkar, Kannan Thiruvengadam, Aarti Kinikar, Lakshmi Murali, Sanjay Gaikwad, Luke Elizabeth Hanna, Vandana Kulkarni, Sathyamoorthy Pattabiraman, Nishi Suryavanshi, Beena Thomas, Rewa Kohli, Gomathi Narayan Sivaramakrishnan, Neeta Pradhan, Brindha Bhanu, Anju Kagal, Jonathan Golub, Neel Gandhi, Akshay Gupte, Nikhil Gupte, Soumya Swaminathan, Amita Gupta, for the CTRIUMPH RePORT India Study Team

**Affiliations:** 1 Byramjee Jeejeebhoy Government Medical College- Johns Hopkins University Clinical Research Site, Pune, Maharashtra, India; 2 Johns Hopkins School of Medicine, Baltimore, Maryland, United States of America; 3 National Institute for Research in Tuberculosis, Chennai, Tamil Nadu, India; 4 Johns Hopkins University–India office (CCGHE), Pune, Maharashtra, India; 5 Byramjee Jeejeebhoy Government Medical College, Pune, Maharashtra, India; 6 District Tuberculosis Officer, State Tuberculosis Office, Thiruvallur, Tamil Nadu, India; 7 Johns Hopkins Bloomberg School of Public Health, Baltimore, Maryland, United States of America; 8 Emory University, Atlanta, Georgia, United States of America; 9 Indian Council of Medical Research, New Delhi, India; Institut de Pharmacologie et de Biologie Structurale, FRANCE

## Abstract

Despite substantial exposure to infectious pulmonary tuberculosis (TB) cases, some household contacts (HHC) never acquire latent TB infection (LTBI). Characterizing these “resisters” can inform who to study immunologically for the development of TB vaccines. We enrolled HHCs of culture-confirmed adult pulmonary TB in India who underwent LTBI testing using tuberculin skin test (TST) and QuantiFERON TB Gold Test-in-tube (QFT-GIT) at baseline and, if negative by both (<5mm TST and <0.35IU/mL QFT-GIT), underwent follow-up testing at 4–6 and/or 12 months. We defined persons with persistently negative LTBI tests at both baseline and followup as pLTBI- and resisters as those who had a high exposure to TB using a published score and remained pLTBI-. We calculated the proportion of resisters overall and resisters with complete absence of response to LTBI tests (0mm TST and/or QFT-GIT <0.01 IU/ml). Using random effects Poisson regression, we assessed factors associated with pLTBI-. Of 799 HHCs in 355 households, 67 (8%) were pLTBI- at 12 months; 52 (6.5%) pLTBI- in 39 households were resisters. Complete absence of response to LTBI tests was found in 27 (53%) resisters. No epidemiological characteristics were associated with the pLTBI- phenotype. LTBI free resisters among HHC exist but are uncommon and are without distinguishing epidemiologic characteristics. Assessing the genetic and immunologic features of such resister individuals is likely to elucidate mechanisms of protective immunity to TB.

## Introduction

Tuberculosis (TB) continues to have a sustained impact on medical and public health systems in low- and middle-income countries (LMIC) and is the leading cause of mortality by an infectious disease pathogen[1]. In 2017, an estimated 10.4 million new TB cases were diagnosed worldwide, and of these, 1.7 million died. Furthermore, 24% of the world’s population (1.7 billion) is estimated to be infected with *Mycobacterium tuberculosis (Mtb)*, representing a large reservoir at risk for progressing to TB disease[2]. To achieve the World Health Organization End TB Strategy targets of 2025, TB incidence should decline by 4–5% per year rather than the current rate of 1.5% per year[2]. Improved socio-economic conditions, nutritional status and living standards can contribute to the reduction of TB, as demonstrated in Western Europe in the past several decades. However, an effective TB vaccine is needed to interrupt TB transmission in LMIC[3,4]. A key rate limiting step for the development of TB vaccines is the limited evidence on immune correlates of protection against acquiring latent *Mtb* infection (LTBI) or developing TB disease.

Household contacts (HHCs) of TB cases are at high risk of acquiring LTBI[5]. The pooled prevalence of LTBI among HHCs ranges from 34.8% (95% CI 27.6–42.7) in high income countries to 52.9% (95% CI 48.9–56.8) in LMIC[5]. Different rates in high and LMIC may be directly related to TB exposure gradients in the household as well as in the community. To identify individuals at highest risk of acquiring LTBI from a TB case, risk scores have been developed, such as the TB contact score by Mandalakas *et al*[6]. Interestingly, despite high exposure to TB cases, a certain number of HHCs never acquire LTBI and are often referred to as “resisters”[5,7–9]. Furthermore, some researchers have postulated that a complete absence of response to LTBI tests may signify not being infected[10,11]. Identifying and characterizing such resisters among HHCs of infectious TB cases is urgently needed to prioritize who to characterize genetically and immunologically to inform the development of TB vaccines[6,10,11].

In this study, we aimed to: a) describe the demographic, clinical, and exposure characteristics of HHCs who remain persistently negative for LTBI at 12 months (pLTBI-) following exposure to culture-confirmed pulmonary TB cases; b) identify HHCs who are resisters (pLTBI-despite a high exposure score), and c) identify a sub-category of resisters who have a complete absence of response to LTBI tests.

## Materials and methods

### Study design and sites

Between August 2014 and December 2017, as part of Cohort for TB Research with Indo-US Medical partnership (C-TRIUMPH) study, two clinical research sites in India—the National Institute of Research in Tuberculosis (NIRT), Chennai, and the Byramjee Jeejeebhoy Government Medical College (BJGMC), Pune, in an academic collaboration with Johns Hopkins University, USA- established a cohort of HHCs of adult pulmonary TB cases[12]. The NIRT site enrolled rural and semi-urban participants attending Poonamalle district and Tiruvallure district TB units of Tamil Nadu. The BJGMC site enrolled particpants attending 11 Revised National TB Control Program (RNTCP) TB units in Pune Municipal and Pimpri-Chinchwad Municipal Corporations of Maharashtra, serving urban and semi-urban populations. Approvals from the ethics committees/ Institutional Review Boards of NIRT, BJGMC and Johns Hopkins University, USA were obtained.

### Study procedures

A HHC was defined as an adult or child living in the same house at any time within the three months prior to an adult (≥18yrs) pulmonary TB index case diagnosis. Following written informed consent, a trained study team collected demographics, medical history, targeted concomitant medication history, and clinical data at entry. All enrolled HHCs also underwent TB symptom screening, anthropometric assessment, and chest radiography at entry, and TB screening and clinical data were collected during follow-up visits at months 4 and 12.

Laboratory investigations included a baseline fasting or random blood glucose test (Cobas c111, Roche Diagnostics Ltd, Switzerland), HbA1c test (BioRad Laboratories Inc, Hercules, CA, USA), HIV testing and sputum evaluation. Spontaneously expectorated sputum from all HHCs was collected on two occasions—on spot and/or early morning. All sputum specimens collected underwent direct smear for acid fast bacilli (AFB) staining, Xpert MTB/RIF assay and cultures (in Mycobacterial Growth Indicator Tube (MGIT) liquid culture and Löwenstein-Jensen (LJ) media) to exclude active TB disease. Tuberculin skin test (TST, Span diagnostics (now Akray), India) was administered as 0.1 ml (5 units) of Purified Protein Derivative (PPD) intradermally on the flexor aspect of the forearm. The reaction was read 48–72 hours later; the size of the reaction was determined by measuring the diameter of induration in millimeters according to standard published methods[13]. QuantiFERON TB Gold Test-in-tube (QFT-GIT, Cellestis) was performed as per manufacturing instructions. In brief, one-milliliter aliquots were collected in three tubes: one with the negative control, one with the positive control and one with TB-specific antigens (ESAT-6, CFP-10, TB7.7). Blood samples were incubated at 37°C ± 1°C overnight. After incubation, the samples were centrifuged and the supernatant removed for ELISA to measure interferon (IFN)-γ concentrations. If the difference in IFN-γ concentration between the TB antigen-stimulated tube and nil tube was ≥0.35 IU/mL and ≥25% of the concentration from the nil tube, the test was defined as positive. A sample was recorded as indeterminate if the difference in IFN-γ concentration between the mitogen tube and nil tube was <0.5 IU/mL or if the nil tube concentration was >8 IU/mL [QFT-GIT package insert]. A repeat TST and/or QFT-GIT was performed at months 4 and 12 if the prior tests were negative.

#### Definitions

For the purposed of this study, LTBI was defined as the presence of a positive TST at ≥5mm or QFT-GIT test result value of ≥0.35IU/mL[13]. Incident LTBI was defined as the newly positive TST of ≥5mm or QFT-GIT test result value of ≥0.35IU/mL within 12 months after study entry among HHCs with negative LTBI at baseline. pLTBI- was defined as negative TST (<5mm, CDC criteria to assess LTBI status) and negative QFT-GIT (<0.35IU/mL) at both baseline and up to 12 months following exposure to the adult index TB case[13]. Those with new QFT-GIT positivity, new TST positive reaction (≥5mm induration) and or active TB disease at follow up were categorized as positive for LTBI. Baseline TB exposure was assessed using a standardized questionnaire developed by Mandalakas et al. In brief, the exposure score is comprised of 11 items, including presence of cough, pulmonary TB, smear positivity of the index case, if the index case was HHC’s primary caregiver or mother, sleep location of the HHC, and whether the HHC lives in the same house as the index TB case[6,9]. High exposure was defined as a score >6 for adults and >5 for children[6,9]. We further defined resisters as HHCs with both pLTBI- and a high TB exposure score. Complete absence of response to LTBI tests among resisters was defined as having 0mm TST and QFT-GIT <0.01 IU/ml at baseline and at all follow-up visits performed by 12 months[10,11]. An additional subcategory of resisters was defined as those with TST 1–4 mm and QFT-GIT 0.01–0.34 IU/mL[10,11]. If only one LTBI test result was available at follow-up, the result of the available test was used to categorize LTBI status using the definitions above.

### Statistical analysis

We used descriptive statistics to assess the proportion of HHCs that were pLTBI-, resisters, and met our definitions of subcategories of resisters based on their TST and QFT-GIT results described above. The conditional distribution of the response given random effects was assumed to be Poisson. To account for clustering within the household, factors associated with pLTBI- were assessed using mixed effects Poisson regression models with random intercept and slope, random effects were assumed to be normally distributed estimates of the random effects models were made using Gauss-Hermite quadrature to approximate the high-dimension integrals that were used in the likelihood. The offset for the Poisson regression was the follow-up time, (i) for resisters: time for screening to end of follow-up and (ii) for TB infection: time from screening to diagnosis of TB infection. All p-values were two-sided with statistical significance evaluated at the 0.05 alpha level. Using the Mandalakas score, we categorized high and low exposure among HHCs. Box plots for QFT-GIT by levels of TST induration were plotted to assess differences in distribution of QFT-GIT by TST. The number of resisters per household examined were plotted using a coral diagram. Statistical analysis was done using STATA 14.2.

## Results

### Prevalence and characteristics of pLTBI- HHCs

**Fig 1:**
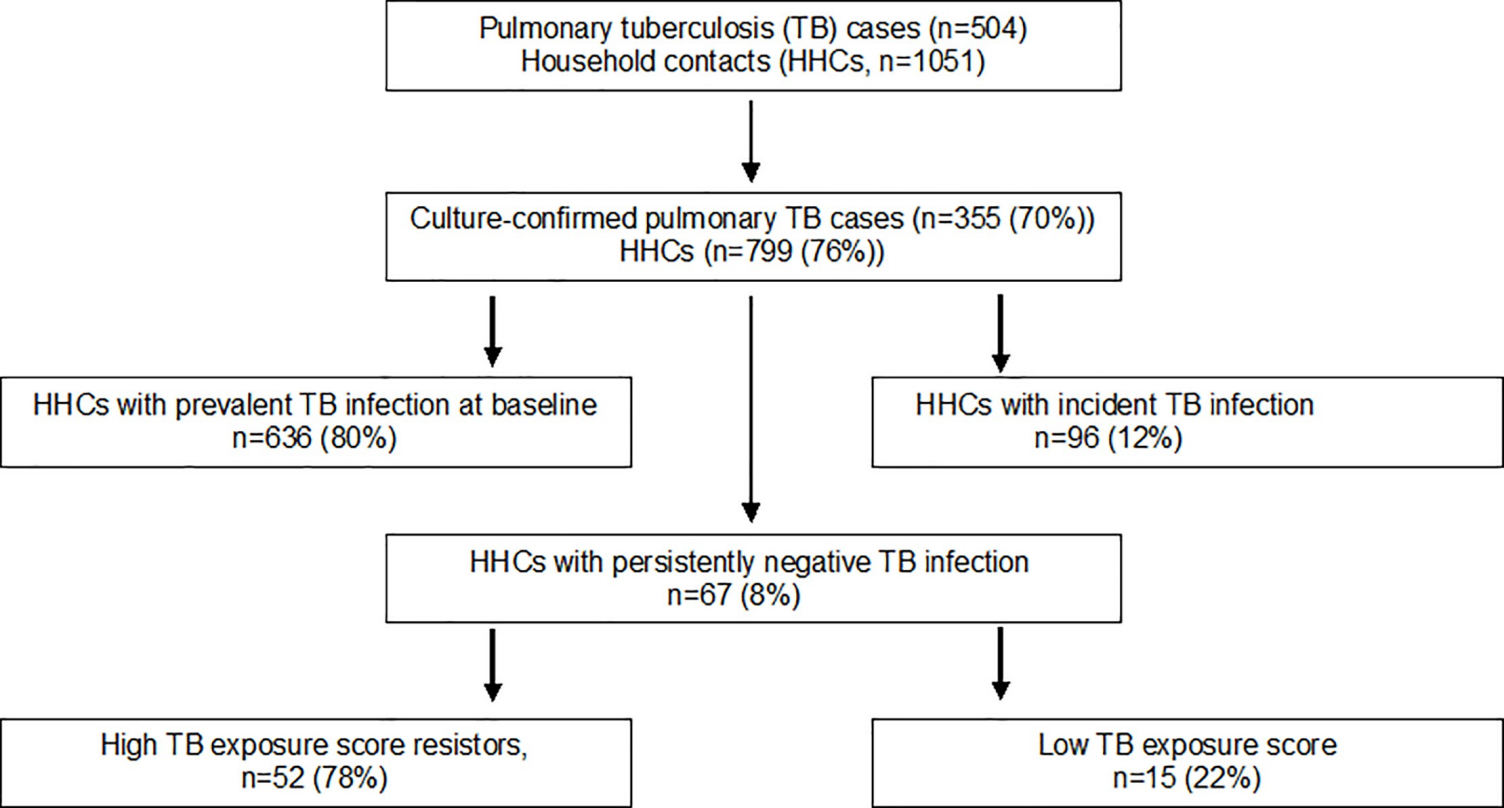
Household contacts with persistently negative latent *Mycobacterial tuberculosis* infection at 12 months (pLTBI-) despite exposure to adults with culture-confirmed pulmonary tuberculosis). Consort diagram showing the number of household contacts with persistently negative latent Mycobacterial tuberculosis infection in 12 months (pLTBI-) despite exposure to adults with culture-confirmed pulmonary tuberculosis.

**Fig 2:**
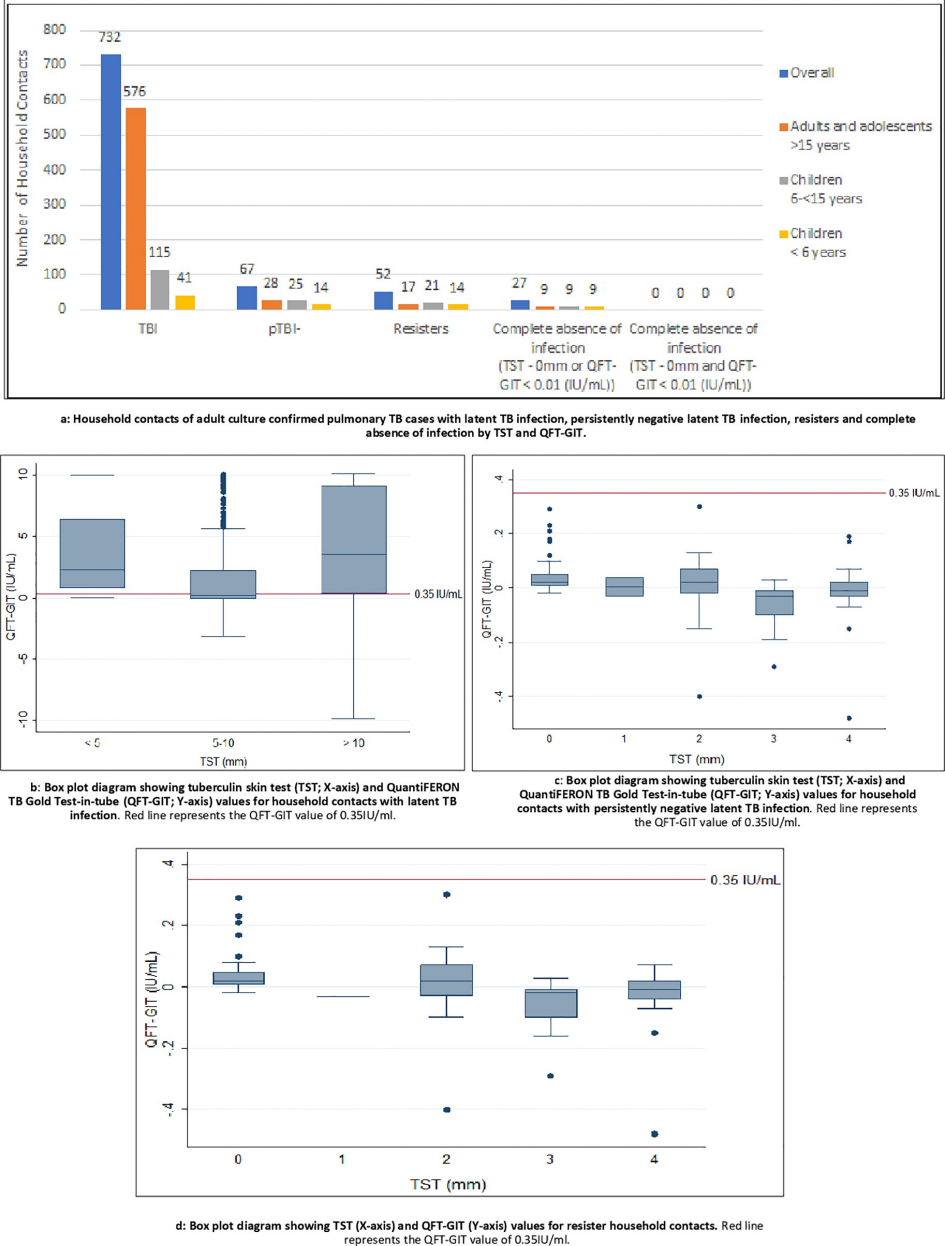
**Figa: Household contacts of adult culture confirmed pulmonary TB cases with latent TB infection, persistently negative latent TB infection, resisters and complete absence of infection by TST and QFT-GIT**. **Fig 2B**: **Box plot diagram showing tuberculin skin test (TST; X-axis) and QuantiFERON TB Gold Test-in-tube (QFT-GIT; Y-axis) values for household contacts with latent TB infection**. **Fig 2C**: **Box plot diagram showing tuberculin skin test (TST; X-axis) and QuantiFERON TB Gold Test-in-tube (QFT-GIT; Y-axis) values for household contacts with persistently negative latent TB infection**. **Fig 2D: Box plot diagram showing TST (X-axis) and QFT-GIT (Y-axis) values for resister household contacts.** Red line in Fig 2B, Fig 2C and Fig 2D represents the QFT-GIT value of 0.35IU/ml.

Of 1051 HHCs enrolled from 504 households, 799 (76%) HHCs from 355 (70%) households were exposed to culture-confirmed pulmonary TB and were included in our analysis. The average number of HHCs per index case was 4, and we enrolled an average of 2.2 HHCs per household. A total of 437 (55%) were female, 604 (75%) were adults and adolescents ≥15years, 55 (7%) were children <6 years, and 140 (18%) were children 6 to ≤14 years. As shown (**Fig 1)**, 636 (80%) from 799 HHCs had prevalent LTBI at entry; 295 (37%) of 780 had both TST and QFT-GIT positivity, 471 (59%) of 799 had only positive TST, and 460 (59%) of 780 had only QFT-GIT positivity. Further, 96 (12%) HHCs developed new LTBI by 12 months, with a total of 732 (99.6%) meeting the definition of LTBI (Fig 2A). Fig 2B shows the distribution of QFT-GIT by different cut-offs for TST among those with LTBI. We had previously reported the overall agreement between TST at 5mm and QFT-GIT, was 60% [kappa = 0.2, 95% CI (0.16, 0.23)][14].

By 12 months, 67 (8%) of 799 HHCs met the definition of pLTBI- Of these 67, 28 (42%) were adults and adolescents and 39 (58%) were children; only 1 had diabetes mellitus and 1 had HIV infection. The household, demographic and clinical characteristics of HHCs and their index cases were not significantly different among HHCs who were pLTBI- and LTBI positive (**Table 1**). The median QFT-GIT values correspondig to TST<5mm was 0.01 (IQR, -0.02–0.03) (Fig 2C).

**Table 1:** Demographic and clinical characteristics and factors associated with persistently negative latent *Mycobacteria tuberculosis* infection among household contacts of culture-confirmed pulmonary TB in India.

Characteristics of HHC	Overall	LTBI^a^	pLTBI-^b^	Relative Risk (95% CI)
	(N = 799)	(N = 732)	(N = 67)	
Age, n (%)				
> 45	120(15%)	119 (16%)	1 (1%)	Ref
15–45	484 (60%)	457 (62%)	27 (40%)	2.94 (0.40–21.63)
Jun-14	140 (18%)	115 (16%)	25 (37%)	4.52 (0.61–33.33)
< 6	55 (7%)	41 (6%)	14 (21%)	5.51 (0.72–41.94)
Sex, n (%)				
Male	362 (45%)	334 (46%)	28 (42%)	Ref
Female	437 (55%)	398 (54%)	39 (58%)	1.09 (0.67–1.77)
Education, n (%)				
Illiterate	99 (13%)	97 (14%)	2 (3%)	Ref
Primary	501 (65%)	455 (65%)	46 (74%)	2.09 (0.51–8.60)
Junior college	112 (15%)	104 (15%)	8 (13%)	1.29 (0.27–6.08)
Graduate	54 (7%)	48 (7%)	6 (10%)	2.29 (0.46–11.34)
Current smoke, n (%)				
No	494 (92%)	472 (93%)	22 (85%)	Ref
Yes	42 (8%)	38 (7%)	4 (15%)	1.38 (0.47–3.99)
Smoking history, n (%)				
Current	36 (6%)	32 (6%)	5 (15%)	Ref
Former	25 (5%)	25 (5%)	0	-
Non-smoker	467 (88%)	445 (89%)	22 (85%)	0.74 (0.25–2.14)
HIV, n (%)				
Negative	427 (98%)	389 (98%)	38 (97%)	Ref
Positive	10 (2%)	9 (2%)	1 (3%)	0.78 (0.11–5.65)
Diabetes mellitus, n (%)				
No DM	748 (94%)	682 (93%)	66 (99%)	Ref
DM	50 (6%)	49 (7%)	1 (1%)	0.36 (0.05–2.62)
BCG scar				
No	84 (37%)	83 (38%)	1 (17%)	Ref
Yes	141 (63%)	136 (62%)	5 (83%)	0.79 (0.09–6.74)
History of isoniazid prophylaxis				
No	431 (99%)	398 (99%)	33 (97%)	Ref
Yes	5 (1%)	4 (1%)	1 (3%)	1.49 (0.20–10.91)
Alcohol, n (%)				
No	434 (81%)	413 (81%)	21 (81%)	Ref
Yes	103 (19%)	98 (19%)	5 (19%)	0.91 (0.34–2.41)
Sleep index, n (%)				
Different building of same household	10 (1%)	8 (1%)	2 (3%)	Ref
Same room, same bed	158 (20%)	147 (21%)	11 (16%)	0.96 (0.21–4.34)
Same room, different bed	355 (45%)	324 (45%)	31 (46%)	0.82 (0.19–3.45)
Same house, different room	257 (34%)	234 (33%)	23 (34%)	0.70 (0.17–3.00)
Other	3 (1%)	3 (1%)	0 (0%)	-
**Characteristics of Index Case**				
Sex, n (%)				
Male	520 (65%)	489 (67%)	31 (46%)	Ref
Female	279 (35%)	243 (33%)	36 (54%)	1.41 (0.86–2.27)
Age, n (%)				
<25	162 (20%)	150 (21%)	12 (18%)	Ref
25–45	361 (45%)	326 (45%)	35 (52%)	1.22 (0.64–2.36)
>45	275 (34%)	255 (35%)	20 (30%)	0.95 (0.46–1.94)
Cavitation, n (%)				
No	313 (46%)	339 (54%)	25 (48%)	Ref
Yes	366 (54%)	288 (46%)	27 (52%)	0.85 (0.50–1.47)
Smear positive, n (%)				
No	254 (32%)	226 (31%)	28 (42%)	Ref
Yes	545 (68%)	506 (69%)	39 (58%)	0.75 (0.46–1.22)
Median time to culture positivity,				
Median (IQR) in days	10 (7–14)	10 (7–13)	11 (7–15)	1.01 (0.97–1.05)
TB cure				
Adverse outcomes	168 (23%)	162 (24%)	6 (10%)	0.74 (0.32–1.72)
**Characteristics of Household**				
Median number of people in HH				
n (IQR)	5 (4–6)	5 (4–6)	5 (4–6)	0.96 (0.89–1.05)
Average monthly income				
<5000	64 (8%)	62 (8%)	2 (3%)	Ref
5000–10000	98 (12%)	88 (12%)	10 (15%)	1.91 (0.41–8.73)
>10000	637 (80%)	582 (79%)	55 (82%)	2.10 (0.51–8.57)
Median number of rooms				
n (IQR)	2 (1–3)	2 (1–3)	2 (2–3)	1.01 (0.99–1.03)
Number of windows				
≥ 2	581 (72%)	539 (74%)	42 (63%)	Ref
< 2	218 (27%)	193 (26%)	25 (37%)	1.16 (0.71–1.91)

Abbreviations: BCG, bacille Calmette-Guerin; HH, household; HHC, household contact; HIV, human immunodeficiency virus; IQR, interquartile range; TB, tuberculosis; LTBI, latent tuberculosis infection; pLTBI-, persistently negative latent tuberculosis infection.

^a^ Defined as new QuantiFERON TB Gold Test-in-tube positivity (>0.35IU/mL), new tuberculin skin test positive reaction (≥5mm induration) and/or active TB disease at follow up.

^b^ Defined as negative tuberculin skin test (<5mm) and negative QuantiFERON TB Gold Test-in-tube (<0.35IU/mL) at both baseline and up to 12 months following exposure to the adult index TB case.

### Exposure characteristics of HHCs who were pLTBI-

There were no significant differences in exposure characteristics between HHCs with pLTBI- or LTBI (**Table 2**). Median exposure score was 5 (IQR,5–6) for pLTBI- compared to 6 (IQR, 5–7) for LTBI positive (p = 0.008) and did not differ significantly among adult or child contacts (p = 0.43, p = 0.08, respectively). Of the 67 with pLTBI-, 39 (58%) were exposed to smear-positive TB cases (of these, 6 (15%) were bedmates), and the remainder was exposed to smear negative, culture positive adult TB cases.

**Table 2:** Exposure characteristics[6] associated with persistently negative latent *Mycobacterium tuberculosis* infection among household contacts of culture-confirmed pulmonary TB cases in India.

Characteristics of HHCs	Overall	LTBI^a^	pLTBI-^b^	Relative Risk (95% CI)
	(N = 799)	(N = 732)	(N = 67)	
Spouse of the index or mother of the child				
No	527 (66%)	474 (65%)	53 (79%)	Ref
Yes	267 (34%)	253 (35%)	14 (21%)	1.05 (0.58–1.89)
Primary caregiver				
No	473 (82%)	450 (82%)	23 (88%)	Ref
Yes	104 (18%)	101 (18%)	3 (12%)	1.07 (0.32–3.59)
Share the same bed				
No	625 (80%)	569 (79%)	56 (84%)	Ref
Yes	158 (20%)	147 (21%)	11 (16%)	1.23 (0.65–2.37)
Sleep in the same room				
No	428 (55%)	392 (55%)	36 (54%)	Ref
Yes	355 (45%)	324 (45%)	31 (46%)	1.05 (0.65–1.70)
Index case coughing				
No	17 (2%)	16 (2%)	1 (1%)	Ref
Yes	781 (98%)	715 (98%)	66 (99%)	0.96 (0.13–6.93)
Index case Smear positivity				
No	254 (32%)	226 (31%)	28 (42%)	Ref
Yes	545 (68%)	506 (69%)	39 (58%)	0.75 (0.46–1.23)
Living in the same household				
No	24 (3%)	23 (3%)	1 (1%)	Ref
Yes	774 (97%)	708 (97%)	66 (99%)	1.86 (0.28–13.39)
Daily meet with index case				
No	25 (3%)	23 (3%)	2 (3%)	Ref
Yes	773 (97%)	708 (96%)	65 (97%)	1.13 (0.28–4.60)
>1 adult TB index case in the household				
No	47 (28%)	41 (27%)	6 (38%)	Ref
Yes	121 (72%)	111 (73%)	10 (62%)	0.75 (0.27–2.05)

Abbreviations: HHC, household contact; TB, tuberculosis; LTBI, latent tuberculosis infection; pLTBI-, persistently negative latent tuberculosis infection.

^a^ Defined as new QuantiFERON TB Gold Test-in-tube positivity (>0.35IU/mL), new tuberculin skin test positive reaction (≥5mm induration) and/or active TB disease at follow up.

^b^ Defined as negative tuberculin skin test (<5mm) and negative QuantiFERON TB Gold Test-in-tube (<0.35IU/mL) at both baseline and up to 12 months following exposure to the adult index TB case.

### Prevalence and characteristics of resisters

**Fig 3:**
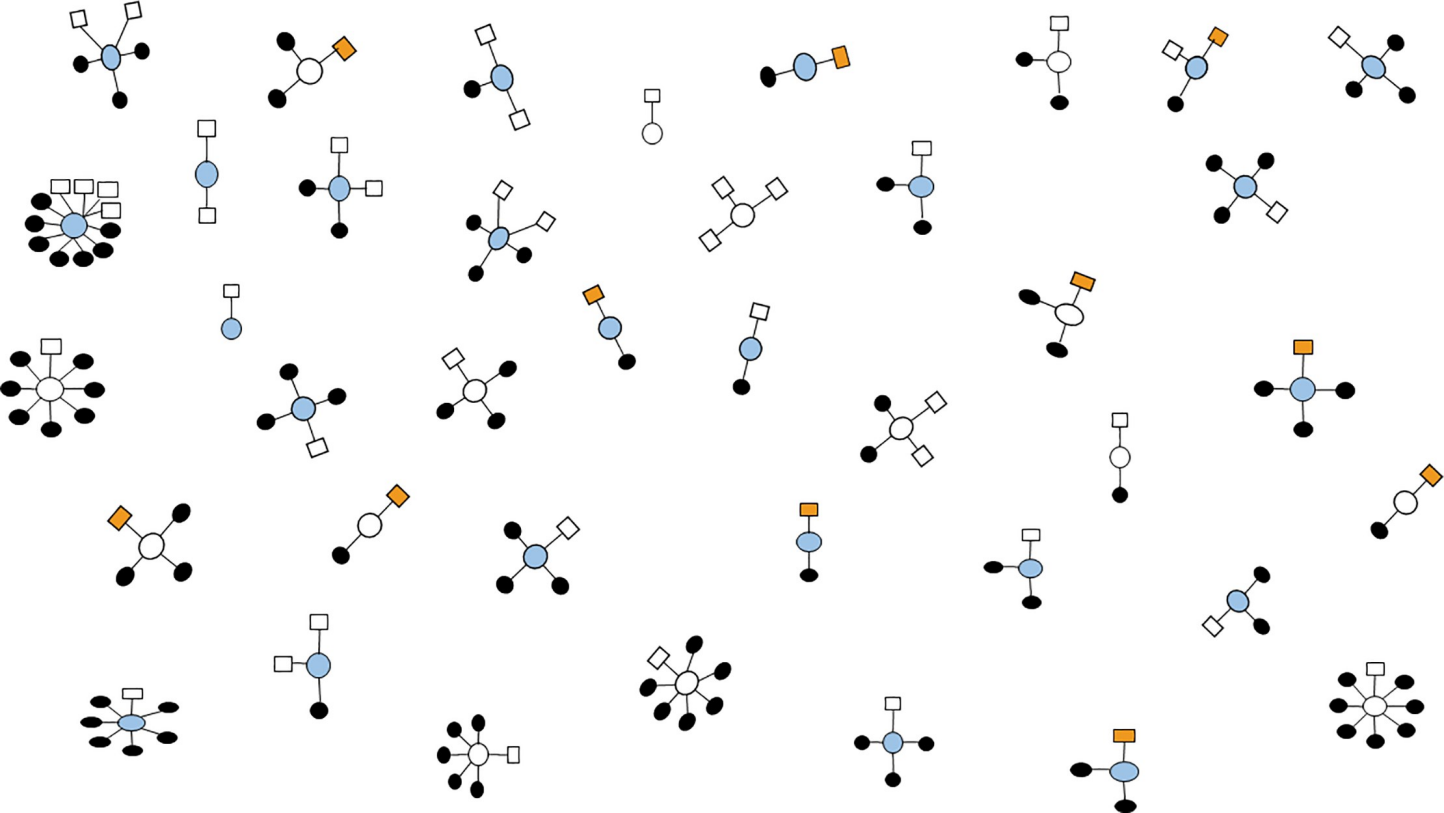
Clustering of household contacts with persistently negative latent *Mycobacterial tuberculosis* infection (pLTBI-) in 39 households stratified by high and low tuberculosis (TB) case exposure is the Fig 3 title. Square represents pLTBI- household contacts with high exposure, referred to as resisters. Orange shaded squares represents household members who share the bed with culture-confirmed pulmonary TB case. Shaded circles represent the household contacts with positive LTBI and blue are smear positive TB cases.

Fifty-two (7%) HHCs with pLTBI- had a high exposure score and met the definition of resisters (**Fig 2D)**; they belonged to 39 (11%) households (**Fig 3)**. The median QFT-GIT values of resisters were 0.01 (IQR, -0.02–0.02). Among the 52 resisters, 17 (33%) were ≥15 years, 21 (40%) were > 6 and ≤ 14 years, and 14 (27%) were <6 years. Thirty-four (62%) were exposed to smear-positive TB cases; of these, 6 (18%) were bedmates. We found that 9 (23%) of the 39 households had 50% or more of their members who were resisters. In 2 households, all HHCs were resisters; the first household had 2 HHCs, a child and an adult exposed to smear-positive TB, and the second household had 3 HHCs, an adult and two children exposed to smear-negative, culture-confirmed PTB.

Twenty-seven (53%) resisters had complete absence of response to at least one of the LTBI tests (either 0mm TST or QFT-GIT <0.01IU/ml at entry and all follow-ups) but none of 15 having both tests completed had absent responses (**Fig 2A, S1 Table)**. Fourteen (27%) resisters were found to have either TST between 1–4 mm or QFT-GIT >0.01 and <0.34 IU/ml.

## Discussion

Our large prospective cohort study assessed epidemiological characteristics of pLTBI- and resisters among HHCs of TB index cases in a high TB burden setting. We found that pLTBI- HHCs are relatively rare and those meeting an epidemiological definition of resister are especially rare. Our analysis did not identify any epidemiological characteristics that could be clearly associated with the pLTBI- or resister phenotype, and none of the resisters had complete absence of response to both TST and IGRA. We did, however, observe clusters of resisters within families living in the same household, Overall, our study adds to the body of evidence supporting the existence of a resister phenotype, at least based on quantified TB exposure and LTBI testing, and our large, well-characterized cohort provides data on a subset of persons who may be resistant to LTBI and will facilitate future research on genomic and immunologic characteristics.

We found low prevalence (7%) of resisters in India using a stringent TST cutoff (<5mm induration as per CDC criteria) and IGRA at baseline then either or both tests at follow-up in the first year after known exposure[13]. In a recent review of 10 historical studies with variable follow-up and exposure settings, Simmons et al reported cumulative resister prevalence ranging from 0% to 35%[15]. More recent studies conducted among HHCs in Uganda and gold miners in South Africa found higher resister prevalence than our study at 12% and 13%, respectively[9,11,15,16]. However, the Uganda studies used a less stringent TST cutoff (<10mm induration) than our study without IGRA. Notably, Stein et al recently completed a longterm follow-up study using a traceable subset of originally enrolled Ugandan HHCs in which IGRA testing and repeat TST were conducted at a median of 9.5 years after known index TB case exposure[17]; of 162 individuals originally found to be persistently negative by TST, 82 remained TST-negative and were found to be IGRA-negative. Interestingly, like our study, many of these prior studies did not identify any distinguishing sociodemographic or epidemiological features that could predict the resister phenotype.

The biological basis of LTBI resistance also remains unknown. In a study that assessed multiple innate and adaptive immunity genes, resistance among Ugandan HHCs was associated with single nucleotide polymorphisms in NOD2, SLC6A3 and TLR4[18]. Unique macrophage- and T cell-mediated mechanisms of LTBI clearance have also been described[15], yet no clear plasma cytokine response was associated with the resister phenotype among Ugandan HHCs. There is renewed interest in TB antibodies and other immune correlates of protection, which are under active investigation. Further, large studies, such as genome-wide association studies, are needed to assess whether there is a genetic signature associated with LTBI resistance. All investigations exploring the biological mechanism of LTBI resistance will need a well-characterized study population of resisters. Clearly, HHCs of TB cases with longitudinal follow-up are an interesting group to study, as there is known exposure that can be quantified as well as family members who likely share multiple genetic determinants[19,20]. Indeed, our study identified several households where 50% of HHCs were resisters and two households where all HHCs were resisters. This group in particular may provide insight into the biologic mechanisms associated with LTBI resistance and warrants further study.

Understanding the biological basis of resistance among children after heavy exposure is also of real interest, as young children are known to be at high risk for progression to TB disease after exposure. Notably, our study is among the few studies that have included children. Like other studies, we found that children are more likely to be pLTBI-, and although not statistically significant, the proportion of pLTBI- HHCs declined as age increased[9,21]. Further, over two-thirds of the resisters in our study were children. Importantly, the use of BCG and complexity of interpreting IGRA results in children need to be considered when applying the resister definition to children,[22] and young children will likely need to be investigated separately from adults, as host-pathogen interactions and mechanisms of infection acquisition and resistance are often different.

Our study has several limitations. Identifying resisters depends on accurate LTBI assessment and TB exposure measurement, yet multiple challenges exist. First, currently available tests do not directly identify LTBI, but instead rely on the host adaptive immune response to *Mtb* proteins. Thus, recently infected individuals who have not yet mounted the required immunological response may not be accurately characterized. Studies have shown that nearly half of HHCs do not test LTBI-positive on initial assessment,[5,7–9] yet up to 20% test LTBI-positive when carefully followed for at least one year[5,23]. Follow-up LTBI testing is important to accurately identify pLTBI-, but requires significant resources to do well. Additional limitations of available LTBI tests that may lead to misclassification of LTBI status include arbitrary TST cutoffs; TST is known to cross react with BCG; and although IGRA overcomes the BCG cross-reaction, both TST and IGRA have known false negativity, false positivity and discordance, which can be as high as 25% in high TB burden countries[14,24–27]. Notably, although our study is among the few to use TST and IGRA to determine LTBI status during follow-up, not all HHCs underwent both tests. Long term follow-up of resisters would be valuable and should be pursued in future studies. Finally, the concordance between TST and QFT-GIT in our study was lower than reports from other research settings, and that could be due to a number of factors, such as lower TB incidence, different BCG coverage, etc. This has implications for future studies that use either or both assays for the definition of LTBI. In a setting with low concordance between the assays, many discordant subjects might be identified and may or may not be considered to be positive for LTBI.

We used ≥5mm for determining TB infection in persons with recent contact with a TB case and this cut-off is based on literature showing a significantly higher risk of active TB at this threshold. The ≥5mm cutoff is the standard that has been used for contact investigation in low incidence settings for over two decades, where contact investigation is a routine part of TB control efforts[13]. In a retrospective study of TB incidence among 33,146 TB contacts in British Columbia, the main risk factors for TB were being a household contact (hazard ratio 8.47) and having a tuberculin skin test (TST) induration of ≥5mm (hazard ratio 4.99). TST induration size was a strong risk marker for TB disease in contacts, even after controlling for other risk factors and prior BCG vaccination[28]. In a retrospective study in Brazil of adolescent and adult HHCs of TB cases, the incidence of TB among 667 contacts was six-fold greater in those with a TST≥5 mm compared to those with a TST<5 mm (5.4% vs. 0.9%; RR 6.04, 95% CI 1.7–20.6). Even among BCG-vaccinated contacts, TST induration of ≥5mm was the only variable that predicted development of TB disease within 2 years[29].

Measuring household TB exposure also poses challenges. We used a published score that lacks extensive validation, but does incorporate key characteristics of index case infectivity and HHC proximity to the index case[6,28]. Interestingly, we found that over one-fifth of HHCs with low exposure scores also had either prevalent or incident LTBI, suggesting community exposure to TB cases. Studies from South Africa and Malawi have shown that only an estimated 8–19% of LTBI can be attributed to household exposure[29]. To identify the best possible study group of resisters, investigators must use advanced epidemiological tools to measure and categorize TB exposure in terms of frequency, location, dose and duration.

## Conclusions

In conclusion, LTBI resisters are relatively uncommon and identification requires large, carefully cultivated cohorts of HHCs with well-characterized exposure measurement, LTBI testing, and longitudinal follow-up. Focusing future research on households that are outliers (i.e. those that lack LTBI despite high exposure) is likely to be the most fruitful approach. In particular, family members in such households should be included in further studies of genetic and immunologic mechanisms of protection against *Mtb* infection acquisition. Specifically for India, which bears the world’s largest TB burden, our well-characterized cohort linked to a specimen repository could facilitate important basic and translational research to accelerate development of novel TB vaccines and further progress towards TB elimination[12].

## Supporting information

S1 Table TST and QFT-GIT results among fifty-two “resisters” household contacts (HHCs) in India. “Resisters” are defined as HHCs with no evidence of LTBI in 12 months following exposure to culture-confirmed pulmonary TB despite high TB exposure (defined as >6 score for adults and >5 score for children using Mandalakas score. *Represents both TST and QFT-GIT was performed at entry, 4 and 12 months among 52 resisters.
(DOCX)
