# Supplementary material for: Infection free “resisters” among household contacts of adult pulmonary tuberculosis
Source: PLoS One. 2019 Jul 18;14(7):e0218034. doi: 10.1371/journal.pone.0218034 (PMC6638997; doi:10.1371/journal.pone.0218034)
Supplement: S1 Table — “Resisters” are defined as HHCs with no evidence of LTBI in 12 months following exposure to culture-confirmed pulmonary TB despite high TB exposure (defined as >6 score for adults and >5 score for children using Mandalakas score. *Represents both TST and QFT-GIT was performed at entry, 4 and 12 months among 52 resisters. (DOCX) [file pone.0218034.s001.docx]

| **Supplementary table 1.** **Tuberculin skin test (TST) and QuantiFERON® TB Gold Test-in-tube (QFT-GIT) results among fifty-two “resisters” household contacts (HHCs) in India** | | | | |
| --- | --- | --- | --- | --- |
| **TST and QFT-GIT characteristic at baseline and follow up** | **Overall**  **N=52** | **Children**  **< 6 years**  **N=14** | **Children**  **6-<15 years**  **N=21** | **Adults and adolescents**  **>15 years**  **N=17** |
| **No response to TST or QFT-GIT** | | | | |
| TST - 0mm or QFT-GIT < 0.01 (IU/mL)  (N=52) | 27 (53%) | 9 (17%) | 9 (17%) | 9 (17%) |
| TST - 0 mm  (N=37) | 13 (35%) | 4 (31%) | 6 (46%) | 3 (23%) |
| QFT-GIT < 0.01 (IU/mL)  (N=44) | 14 (32%) | 5 (36%) | 3 (21%) | 6 (43%) |
| *TST - 0mm and QFT-GIT < 0.01 (IU/mL)  (N=15) | 0 | - | - | - |
| **Other resisters** | | | | |
| TST - 1mm - 4mm or QFT-GIT - 0.01 IU/mL - 0.34 IU/mL  (N=52) | 14 (27%) | 4 (29%) | 4 (29%) | 6 (42%) |
| TST - 1mm - 4mm  (N=37) | 8 (22%) | 3 (38%) | 1 (12%) | 4 (50%) |
| QFT-GIT - 0.01 IU/mL - 0.34 IU/mL  (N=44) | 6 (14%) | 1 (17%) | 3 (50%) | 2 (33%) |
| *TST - 1mm - 4mm and QFT-GIT - 0.01 IU/mL - 0.34 IU/mL  (N=15) | 1 (2%) | - | - | 1 (100%) |
| “Resisters” are defined as HHCs with no evidence of LTBI in 12 months following exposure to culture-confirmed pulmonary TB despite high TB exposure (defined as >6 score for adults and >5 score for children using Mandalakas score [6])  *represents both TST and QFT-GIT was performed at entry, 4 and 12 months among 52 resisters. | | | | |
